# A Preliminary Study for Reference RF Coil at 11.7 T MRI: Based on Electromagnetic Field Simulation of Hybrid-BC RF Coil According to Diameter and Length at 3.0, 7.0 and 11.7 T

**DOI:** 10.3390/s22041512

**Published:** 2022-02-15

**Authors:** Jeung-Hoon Seo, Jun-Young Chung

**Affiliations:** 1 Neuroscience Research Institute, Gachon University, Incheon 21988, Korea; jeunghoon.seo@gachon.ac.kr; 2 Department of Neuroscience, College of Medicine, Gachon University, Incheon 21565, Korea

**Keywords:** reference radiofrequency coil, hybrid-type birdcage coil, finite difference time-domain method, FDTD method, electromagnetic field simulation, magnetic resonance imaging, 11.7 T MRI

## Abstract

Magnetic resonance imaging (MRI) systems must undergo quantitative evaluation through daily and periodic performance assessments. In general, the reference or standard radiofrequency (RF) coils for these performance assessments of 1.5 to 7.0 T MRI systems have been low-pass-type birdcage (LP-BC) RF coils. However, LP-BC RF coils are inappropriate for use as reference RF coils because of their relatively lower magnetic field (B_1_-field) sensitivity than other types of BC RF coils, especially in ultrahigh-field (UHF) MRI systems above 3.0 T. Herein, we propose a hybrid-type BC (Hybrid-BC) RF coil as a reference RF coil with improved B_1_-field sensitivity in UHF MRI system and applied it to an 11.7 T MRI system. An electromagnetic field (EM-field) analysis on the Hybrid-BC RF coil was performed to provide the proper dimensions for its use as a reference RF coil. Commercial finite difference time-domain program was used in EM-field simulation, and home-made analysis programs were used in analysis. The optimal specifications of the proposed Hybrid-BC RF coils for them to qualify as reference RF coils are proposed based on their B_1_^+^-field sensitivity under unnormalized conditions, as well as by considering their B_1_^+^-field uniformity and RF safety under normalized conditions.

## 1. Introduction

In magnetic resonance imaging (MRI) devices below 7.0 T, birdcage (BC) radiofrequency (RF) coils are widely used for the production of a circularly polarized mode in quadrature current driving [1,2]. Because the BC coil configuration provides superior magnetic flux density field (B_1_-field) sensitivity and uniformity, this BC geometry has been widely applied to MRI fields requiring uniform RF transmission field (B_1_^+^-field) and RF reception field (B_1_^−^-field) instead of other RF coil geometries [3]. For these advantages, BC coils are usually used as reference RF coils when performing quality assurance testing in monthly system checks as well as in image quality checks, especially when used to validate quantitative performance. In particular, in clinical MRI systems under 7.0 T, quantitative performance evaluations are usually performed using a low-pass-type BC (LP-BC) RF coil. In clinical settings, to obtain high-field (HF) and ultrahigh-field (UHF) MRI signals and replace existing coil-based structures for low-field (LF) MRI, the demand for the development of new structures and electronic devices is increasing [4].

However, LP-BC RF coils are characterized by performance limitations in terms of their signal sensitivity in ultrahigh-field (UHF) MRI systems above 3.0 T. In fact, LP-BC RF coils provide excellent B_1_-field sensitivity in low-field MRI systems below 3.0 T, and their B_1_-field sensitivity decreases significantly as magnetic field strength increases above 3.0 T compared to other types of BC RF coils [5,6]. Despite the low signal sensitivity problem of LP-BC RF coils, MRI manufacturers perform the aforementioned evaluation on 3.0 and 7.0 T MRI systems using LP-BC RF coils as reference coils. However, it would be preferable to use other types of BC RF coils with higher signal sensitivity than LP-BC RF coils in MRI above 7.0 T. To resolve these performance limitations in terms of low B_1_-field sensitivity, both the undertaking of research into different types of BC coils and the obtaining of an optimized structure for such coils are essential [7]. This study proposes the optimal dimensions of the BC RF coil as 3.0, 7.0 and 11.7 T to determine the optimal dimensions of the reference RF coil for the newly constructed and installed 11.7 T human MRI system.

Previous studies have proposed the use of certain RF coils following the undertaking of performance comparisons under limited or specific conditions and after considering signal sensitivity, uniformity, or specific absorption rate (SAR) safety—each parameter on its own—based on the various structures of available RF coils. For example, improved B_1_^+^-field homogeneity is observed in surface-type RF coils that provide high signal sensitivity near the RF coil and multichannel RF coils for parallel imaging [8]. The structure of the surface RF coils was evaluated through the performance analysis of the RF coils in terms of signal sensitivity [9,10]. Volume-type RF coils (such as the BC RF coils) have been mainly assessed in terms of their field uniformity [11,12]. In addition, RF safety has been considered in terms of SAR safety for clinical application [13,14]. However, to propose an RF coil, the performance evaluation of the latter should be performed in a comprehensive manner based on the simultaneous consideration of signal sensitivity, field uniformity, and SAR safety.

BC RF coils are classified into three types (low-pass, high-pass, and hybrid coils) depending on the location of the capacitor attached to the legs and end-rings [15]. An LP-BC RF coil can reduce the unit cost and can improve its quality due to the advantages of using a small quantity of required capacitors and fewer capacitance values [16,17]. The high-pass-type BC (HP-BC) and hybrid-type BC (Hybrid-BC) RF coil configurations exhibit higher |B_1_|-field sensitivity than the LP-BC RF coil at various magnetic field (B_0_-field) strengths [5,18]. In fact, the HP-BC RF coil exhibited better |B_1_|-field sensitivity than the other configurations, whereas the |B_1_|-field sensitivity of the LP-BC RF coil decreased dramatically with the increase in the B_0_-field strength. On the other hand, the Hybrid-BC RF coil provides the highest B_1_-field sensitivity and homogeneity when used at UHF MRI systems over 7.0 T [5]. In addition, the diameter (D) and length (L) of the BC RF coils determine B_1_-field sensitivity and uniformity; thus, these coils are designed to operate in close proximity to the image acquisition target. The BC RF coils are typically set to a diameter-to-length ratio (D/L-ratio) of 1.0, which is the Helmholtz condition for having an equal loop radius as the distance between the two loops. A D/L-ratio of 1.0 provides high B_1_-field uniformity, whereas the D/L-ratio must be set to 0.7 to ensure maximum sensitivity at the middle plane [15]. However, the B_1_-field inhomogeneity effect usually appears as a bright region in the middle of the image when the B_0_-field strength is set at over 3.0 T. Despite these disadvantages, studies on Hybrid-BC RF coils operating at 3.0 T and above have been steadily conducted to address several changes in B_1_^+^-field sensitivity in the traverse plane as well as in the peak uniformity in different locations along the traverse plane [19,20]. The Hybrid-BC RF coil provides low sensitivity to loading (electromagnetic balance) with lossy dielectric loads, and when combined with carefully selected leg capacitor and ring capacitor ratios, it can reduce frequency sensitivity to loading by canceling the leg and ring effects [17,21].

In UHF MRI systems, BC RF coils demonstrate strong B_1_-field nonuniformity mainly due to a shortened electromagnetic wavelength generated at a high frequency. The high electrical permittivity of the water molecules in the human body further shortens the wavelength by causing standing wave patterns [22,23]. In addition, the electrical conductivity of the tissues attenuates the electromagnetic field (EM-field) energy induced by the RF coil. The high permittivity and conductivity of the human tissues cause the severe nonuniformity of the B_1_-field in UHF MRI systems with a B_0_-field strength of 7.0 T or higher [24,25]. In terms of RF safety, the SAR value is an important factor. In particular, an EM-field simulation is essential to more accurately predict the safety of RF coils as EM-fields play an important role in the generation of B_1_-fields and SAR distributions [26,27,28,29,30,31,32,33,34,35,36,37,38,39,40,41,42,43,44,45,46,47,48,49,50,51,52,53,54,55,56,57]. Owing to these problems, various studies have been conducted with an aim to increase the uniformity of the B_1_-field [26,27,28,29,30,31,32,33,34,35,36,37] and to improve the sensitivity of the B_1_-field [38,39,40,41,42,43,44,45] along with RF safety [46,47,48,49,50,51,52,53,54,55,56,57].

In this study, we herein propose the optimized diameter and length of the Hybrid-BC RF coil when considering it as a reference coil for the assessment of the performance of the 11.7 T MRI system that is currently being developed by Gachon University Gil Medical Center (GUGMC, Incheon, Korea). Moreover, it was also performed to define the type and size of Hybrid-BC coils that provide better signal sensitivity and uniformity in 3.0 T, 7.0 T and 11.7 T MRI systems. To suggest an optimal BC coil for each magnetic field strength, we have comprehensively investigated the use of a Hybrid-BC RF coil as a reference RF coil by utilizing a cylindrical phantom model (made of distilled water) and human body model under various B_0_-field strengths (at 3.0, 7.0 and 11.7 T) and by considering signal sensitivity, field uniformity, and SAR analysis. EM-field simulations were performed to analyze the magnitude maps (|B_1_^+^|-field) for the assessment of the generated EM-field sensitivity, as well as their phase maps for the assessment of the generated EM-field uniformity. Moreover, SAR analysis was performed in terms of RF safety.

## 2. Materials and Methods

### 2.1. EM-Field Simulation Setup

To evaluate the EM-field distribution of the Hybrid-BC RF coil with regard to the changes occurring in its B_0_-field and D/L-ratio, we accessed the generated |B_1_|-field distribution using the commercially available EM-field simulation software Sim4Life™ v4.4 (Zurich MedTech AG, Zurich, Switzerland). This EM-field simulation program is widely used in advanced computational calculations. It uses Yee cells in the same way as the finite-difference time-domain (FDTD) method uses them [58]. Three-dimensional Yee cells were adopted along the *x*, *y*, and *z* axes with a 1 mm resolution (including the absorbing boundary condition with a perfect match layer for the acquisition of accurate B_1_-field distribution) and −70 dB conversions to produce a steady-state equilibrium condition for the studied Hybrid-BC RF coil. Current sources located at the legs and end-rings of the coil were used as a substitute for capacitors, and phase information was set according to the geometrical structure position information. The current sources comprised harmonic excitation signals with an amplitude of 1 A and were used assuming an ideal condition in which uniform RF signals were applied to the coils. The current source operates under conditions in which the RF applied from the RF source (transmission port) is ideally driven by a micro-strip line composed of perfect electric conductor (PEC). The current intensity and phase of the RF can be defined at each current drive point replacing the capacitor, and the EM-field can be obtained at a specific resonance frequency. This simulation was performed under the assumption that the current was ideally drive by the micro-stripe line of the BC RF coil [5,9,19,28,32,36,44]. The results of the FDTD complex data matrix were computed using MATLAB (version 2020a, Mathworks, Inc., Natick, MA, USA) for the positioning of the B_1_-, B_1_^+^-, and B_1_^−^-components within their phase map and for the comparison of the electric field (E-field) and SAR components under various B_0_-field strengths. Finally, to identify changes in the B_1_-field resulting from the increase in the B_0_-field strength, EM-field simulations were conducted at 127.74, 300, and 500 MHz, which translate to Larmor frequencies (λ) of 3.0, 7.0, and 11.7 T, respectively.

Each of the EM-fields was categorized in two groups: those meeting the unnormalized condition and those meeting the normalized condition. The unnormalized case provides a comparison of EM-field sensitivity with the EM characteristic changes of each of the coil’s geometries under the same conditions, and the normalized case intends to predict the actual experimental conditions when a π/2 RF pulse was induced, in a similar manner to that of the MRI experiments. EM-field sensitivity was assessed by employing the evaluation criteria using the centerpoint (CP) value, and mean value under the unnormalized condition. The sensitivity of the |B_1_^+^|-field was assessed using the normalization factor required to assume π/2 RF excitation. EM-field uniformity was evaluated using the standard deviation (STD) value under the normalized condition. Normalized SAR calculation is proposed in this paper as an indicator that allows us to predict the safety of real MRI experiments.

**Figure 1:**
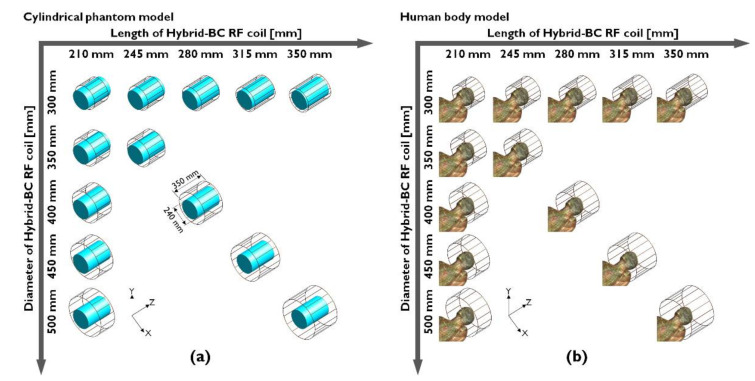
Geometric models of the 16-leg Hybrid-BC RF coils with various diameters and lengths generated using a cylindrical phantom model (**a**) and a human body model (**b**) for the undertaking of the finite-difference time-domain calculation.

The numerical calculation results indicated that the B_1_-field distributions of the Hybrid-BC RF coils provided different results with the increase in the applied B_0_-field strength. The EM-field simulation results suggest that before determining the D/L-ratio of the RF coils, the operational resonance frequency based on B_0_-field strength and dielectric properties should be considered along with the EM characteristics of the object. To analyze the characteristics of the Hybrid-BC RF coil as it changes in terms of its diameter and length, the geometries of the Hybrid-BC RF coil were modeled in two ways: (i) with diameter fixation allowing for an increase in length and (ii) with length fixation allowing for an increase in diameter. Furthermore, we built a third category of simulation models with parallel length and diameter adjustments so that the D/L-ratio in each instance was set at 0.7. In Figure 1, the Hybrid-BC RF coils were modeled with varying diameters and lengths using three cases: (i) a case in which the diameter increased from 300 to 500 mm; (ii) a case in which the length increased from 210 to 350 mm; and (iii) a case in which both the diameter and length increased while maintaining a D/L-ratio of 0.7. The Hybrid-BC RF coil was configured using a perfect electric conductor with 16 legs. The configuration of the cylindrical phantom model (using distilled water) was set at a diameter of 240 mm and length of 350 mm. The cylindrical phantom model was located at the center of the coil and comprised distilled water with specific parameters of relative permittivity (ε_r_ = 76.7) and conductivity (σ = 5 × 10^−5^ S/m), as defined for distilled water in the material database provided by Sim4Life™ v4.4. On the other hand, the dielectric properties of the human body model (DUKE phantom by IT’IS Foundation, Zurich, Switzerland) were also defined in Sim4Life™ v4.4 (Zurich MedTech AG, Zurich, Switzerland) [59].

In this study, computational calculations were performed on a workstation for EM-field simulation with GPU hardware acceleration. The simulation computer was constructed using an Intel Xeon CPU (E5-2640 v4 @ 2.40 GHz) with a 256 GB RAM. For Compute Unified Device Architecture (CUDA) operations, an NVDIA Titan XP graphics card (D5X 12 GB) was used on a 64-bit Windows 10 Pro operating system.

### 2.2. Analysis

The EM-field analysis of the Hybrid-BC RF coil was performed on B_1_^+^-fields with their phase maps, and these fields are, in fact, the transmission components of the B_1_-field, respectively. In this numerical analysis, the |B_1_^+^|-field and |B_1_^−^|-field distributions were calculated using the reciprocity theorem [60]. More specifically, the |B_1_^+^|-fields were compared based on their performances under two conditions: the unnormalized and normalized conditions. In this paper, we calculated the |B_1_^+^|-field distributions within their respective phase maps (B_1_^+^-field), as well as the SAR maps, under the unnormalized condition. In the obtained normalized |B_1_^+^|-field results, the central pixel of the BC coil in the |B_1_^+^|-field maps was calculated by assuming that the flip angle was π/2, and thus, the RF pulse was normalized at 1.957 μT. This normalization factor corresponds to a π/2 flip angle with a 3 ms rectangular RF pulse [61,62]. It also indicates the signal sensitivity of the unnormalized |B_1_^+^|-field.

From the results of the EM-field simulation, phase maps can be obtained using the *x*-, *y*-, and *z*-components of each calculated |B_1_|-field [57]. The components of $B_{1x}$, $B_{1y}$, and $B_{1z}$ are defined as follows [63]:

$$B_{1x}=-B_{0}\sin\alpha \cos2\alpha$$

$$B_{1y}=B_{0}\sin2\alpha$$

$$B_{1z}=B_{0}\cos\alpha \sin2\alpha$$

Here, $B_{1x}$, $B_{1y}$, and $B_{1z}$ are the B_1_-field components (same as in magnetization) of the *x*-, *y*-, and *z*-axes, respectively. The phase of the transverse component $B_{xy}$ can be defined as follows:

$$B_{1z}=B_{0}\cos\alpha \sin2\alpha$$

Finally, the phase map calculation equation can be written as

$$B_{1\_phase}=\mathrm{angle}\left(B_{1,x{\_}_{component}}\right)+i(\mathrm{abs}(B_{1,y{\_}_{component}}))$$

The phase of the B_1_-field is denoted as $B_{1\_phase}$. The $B_{1,x{\_}_{component}}$ and $B_{1,y{\_}_{component}}$ are the *x*- and *y*-components of the B_1_-fields as calculated from the EM-field simulations.

The phase maps of the B_1_-, B_1_^+^-, and B_1_^−^-fields were calculated and analyzed using these numerical processes. Using the acquired EM-fields and their phase maps, the simulations were analyzed in two ways: one for the unnormalized cases and one for the normalized cases. The |B_1_^+^|-field with the SAR map was calculated under the unnormalized condition, and the |B_1_^+^|-field with SAR map was numerically recalculated under the normalized condition using the normalization factor as calculated in the |B_1_^+^|-field. During B_1_^+^-field excitation using an RF pulse, the E-fields that are produced in the conductive tissues can generate electrical currents into the tissues and can cause tissue heating. This tissue heating can cause serious side effects and should be prevented [40]. The most widely used method for evaluating these side effects is to identify SAR distribution.

To evaluate and predict the impact of EM waves on humans in terms of RF safety, the SAR distributions of the EM-field simulation are an important evaluation factor. This is particularly important as it is difficult to measure the actual SARs in the human body. In particular, the EM-field simulation is essential to more accurately predict the safety of the RF coils as the E-fields play an important role in the generation of B_1_-fields and SAR distribution. SAR values provide an estimate of the temperature rise caused in the tissue, and they are calculated as the ratio between the dissipated power and mass density. In this paper, the SAR values were calculated using local SAR values [64,65,66].

The energy transmitted by a time-harmonic EM-field is accumulated in lossy objects by inducing conduction currents. The power loss is denoted as L, and it was produced by the conduction current σΕ results. It is numerically computed by integrating the power density within the lossy volume:

$$L=\frac{1}{2}\int \sigma Ε\cdot {Ε}^{*}dv$$

where $Ε=Ε\left(r,\omega \right)$ is the time-harmonic electric field phasor (V/m), ${Ε}^{*}$ is the conjugate transpose of Ε, and $\sigma =\sigma \left(r\right)$ is the electric conductivity (1/Ωm) at every location $r=\;\left(x,y,z\right)$.

In EM dosimetry, the amount of energy absorbed by the biological tissue is expressed in SARs. The local SAR can be expressed as

$$\mathrm{SAR}\left(r\right)=\frac{\sigma }{2\rho }{Ε}^{2}\propto \frac{dT}{dt}$$

where ${Ε}^{2}=Ε\cdot {Ε}^{*}$ denotes the squared magnitude of the induced electric field, $\rho =\rho \left(r\right)$ is mass density (kg/m^3^), and T is temperature (K).

In this study, the SAR values were analyzed using local SAR values, which are defined as unnormalized SAR. In the EM-field simulation of the RF coils for MRI, the local SAR values were calculated using the |E|-fields that occurred when the current drives through the RF coil without specifying the strength of the applied RF signal. However, in this case, the local SAR values cannot be quantitatively evaluated based on the geometries of the RF coils. In an attempt to solve this problem, the application of a normalization factor has been assumed to stimulate the |B_1_^+^|-fields by π/2 excitation to the calculated unnormalized SAR values, thus allowing for a relative comparison of the SAR values when applying a π/2 RF pulse. These normalized SAR values are simply calculated by applying the normalization factors to unnormalized SAR values. To predict the effects of the Hybrid-BC RF coils on the human body model, a SAR analysis was conducted, and the study of the SARs after the normalization factor was applied was proposed as a more (suitable) quantitative SAR analysis method. Depending on the B_0_-field strength changes and different dimensions of the Hybrid-BC RF coil, the EM characteristics of the Hybrid-BC RF coil are presented in this paper under the unnormalized and normalized conditions.

## 3. Results and Discussion

**Figure 2:**
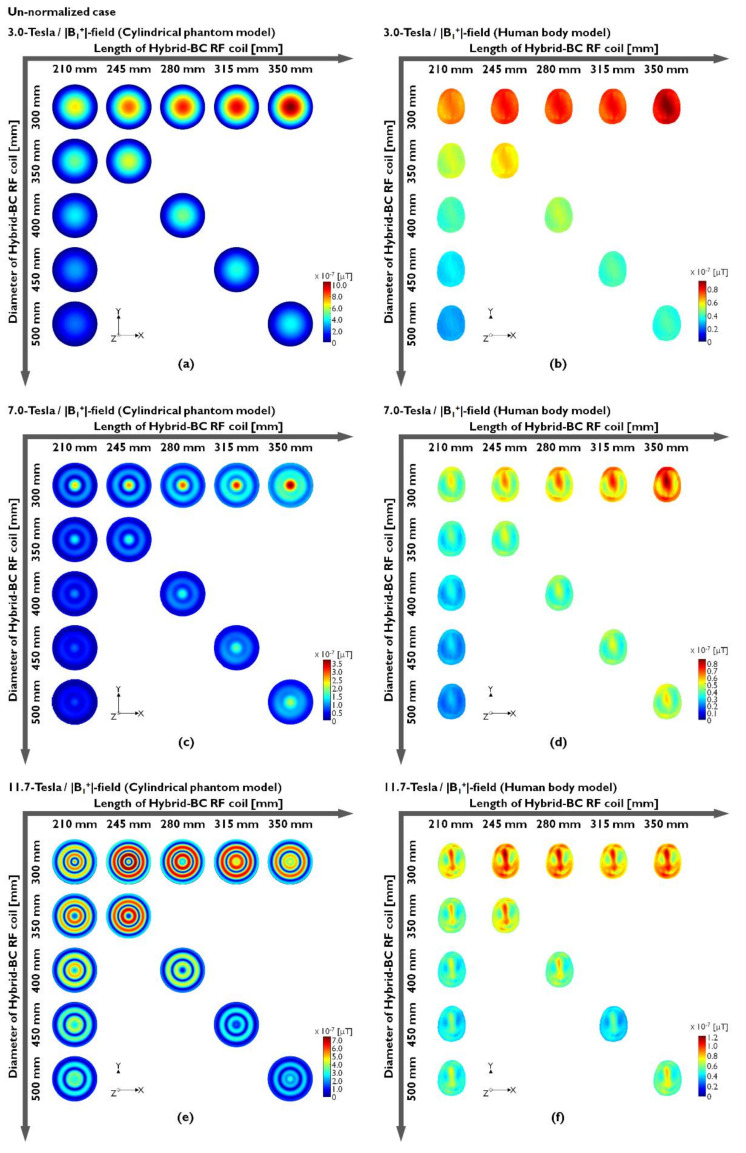
Unnormalized |B_1_^+^|-field distribution obtained when using a cylindrical phantom model (**a**,**c**,**e**) or a human body model (**b**,**d**,**f**).

Hybrid-BC RF coils were compared along the center slice in the axial direction of the unnormalized and normalized conditions. To evaluate the sensitivity of the B_1_^+^-field, we analyzed both the magnitude maps (|B_1_^+^|-field) and its phase map to evaluate the field uniformity generated with increasing B_0_-field strength. The unnormalized |B_1_^+^|-field results of the cylindrical phantom model made using distilled water at 3.0 T (127.74 MHz), 7.0 T (300 MHz), and 11.7 T (500 MHz) are provided in Figure 2a,c and Figure 2e, respectively. Moreover, the results of the human body model are presented in Figure 2b,d,f. The unnormalized |B_1_^+^|-field results are also presented numerically in Table 1 and in Figure 2.

In the EM-field simulation results using the water phantom, the unnormalized |B_1_^+^|-field sensitivity at 127.74 MHz was verified by an increase in parallel to the length increases, and the diameter of the Hybrid-BC coil is fixed at 300 mm (Figure 2a). The latter means that the sensitivity of the unnormalized |B_1_^+^|-field improved as the coil length increased and the diameter was fixed at 300 mm. At 300 MHz with the resonance frequency at 7.0 T, the unnormalized |B_1_^+^|-field results that were analogous to 3.0 T can be observed in Figure 2c, but there has been an inhomogeneity in which the RF fields appear concentrated in the center point of the phantom. The results of 11.7 T showed that the RF fields were concentrated similarly to rings to the peripheral region rather than to the center of the phantom (Figure 2e). In actual MRI experiments, this ring-shaped inhomogeneity field pattern is expected to make it difficult to obtain 11.7 T MR images with a cylindrical phantom model made of distilled water. These results are caused by the dielectric properties of distilled water and the structure of the uniform phantom. Because of this nonuniformity, practical experiments using 7.0 T MRI require conducting uniformity tests using oil phantoms. The unnormalized |B_1_^+^|-field simulation results using human body models tended to be more analogous in their simulation results than the results obtained using water phantoms in Figure 2b,d,f, and ring-shaped imbalance was not confirmed at 11.7 T. According to the reciprocity theorem, the transmission B_1_^+^-field contributes to the RF flip angle whereas the reception B_1_^−^-field contributes to the received signal [67]. The Hybrid-BC RF coils have |B_1_^−^|-field close to zero (Figure S1 and Table S1), which affect the net |B_1_|-field during low-field MRI. As observed from Figure 2, the accurate transmission of RF signals to the center of the object may not be possible while using 11.7 T MRI.

**Figure 3:**
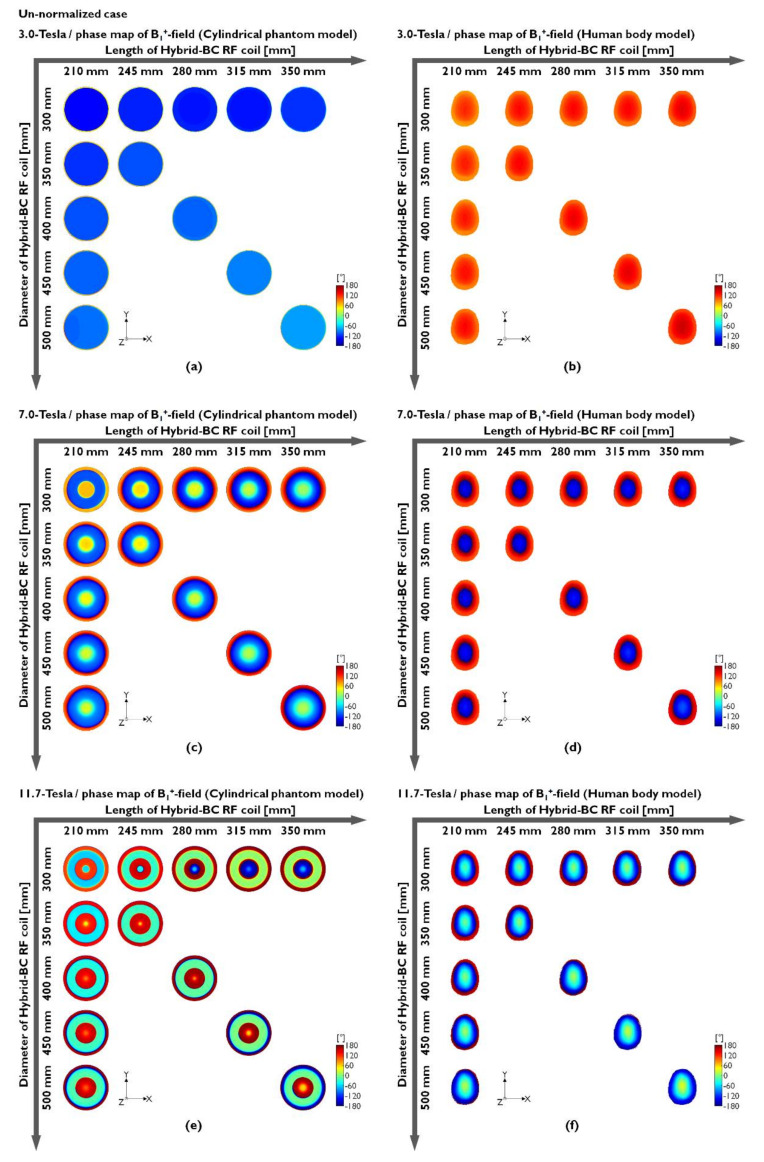
Phase map of the unnormalized B_1_^+^-field distribution obtained when using a cylindrical phantom model (**a**,**c**,**e**) or a human body model (**b**,**d**,**f**).

In Table 1, the unnormalized |B_1_^+^|-field values were verified by CP and mean values. Table 1 shows the intensity values of the results presented in Figure 2. The CP values were increased as the length increased and decreased as the diameter increased. However, the mean value also demonstrated a reduced effect, thus reducing the overall field sensitivity. The phase map of the unnormalized B_1_^+^-field was calculated from the x- and y-components of the unnormalized B_1_^+^-field as shown in Figure 2. As with the unnormalized |B_1_^+^|-field results presented in Figure 2, the results of the phase map obtained using a cylindrical phantom model made of distilled water were also identified to be inhomogeneous within the rotational phase at 7.0 T (Figure 3c) and 11.7 T (Figure 3e).

Figure 3a,b shows the phase maps of the cylindrical phantom model (using distilled water) and human body model at 3.0 T, respectively. It is easily observed that the phase maps were relatively uniform compared to the results obtained at 7.0 and 11.7 T, but the field distribution was not completely uniform. This means that the unnormalized |B_1_^+^|-field inhomogeneity occurs because of the 3.0 T cylindrical phantom model results. Moreover, the human body model results presented with analogous nonuniform phase maps at 7.0 T (Figure 3d) and 11.7 T (Figure 3f). In fact, the 7.0 T results showed that the phase map was rotated and distorted once, whereas the phase map was rotated and distorted twice in the case of 11.7 T. As shown in Figure 3f, the 11.7 T simulation results using the human body model were not observed with extreme phase map nonuniformity compared to those of the cylindrical phantom model made of distilled water. As can be predicted by these results, the main factor able to determine |B_1_^+^|-field uniformity is the phase distortion of the B_1_-field as a result of the RF’s shortened EM wavelength, the size of the phantom, and its dielectric properties at the UHF. The unnormalized |B_1_^+^|-field and its phase maps presented in Figure 2 and Figure 3 tended to present with an analogous field distribution as compared to the unnormalized |B_1_|-field simulation results presented in Figure S3 and Table S2. The distortions in the phase map were confirmed not only in the phase map of the |B_1_^+^|-field but also in the phase map distribution of the |B_1_^−^|-field and |B_1_|-field in Figures S2 and S4.

As confirmed by the simulation results, the unnormalized |B_1_^+^|-field determines the sensitivity of the unnormalized |B_1_|-field. On the other hand, it was confirmed that the distortion of the unnormalized B_1_^−^-phase affects the uniformity of the unnormalized |B_1_|-field. In summary, the unnormalized |B_1_^+^|-field with a B_1_^+^-phase map is associated with the sensitivity of the unnormalized |B_1_|-field, and the phase map of the unnormalized B_1_^−^-field is related to the uniformity of the unnormalized |B_1_|-field.

**Figure 4:**
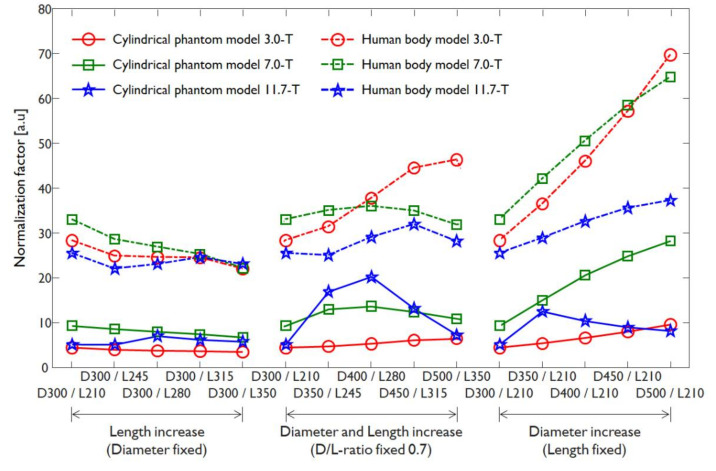
Normalization factors applied for the normalized |B_1_^+^|-field calculation when using a cylindrical phantom model or a human body model at 3.0, 7.0, and 11.7 T MRI systems.

The normalized EM-field was assumed by the normalization factors calculated when using the EM-field simulation results under the unnormalized conditions presented in Figure 2. Here, the π/2 flip angle with a 3 ms rectangular RF pulse is assumed a π/2 RF pulse; thus, it was normalized to 1.957 μT. The normalization factor represents the amount of RF energy required when a π/2 RF pulse is applied, and it is used to measure the signal sensitivity of the unnormalized |B_1_^+^|-field. The unnormalized |B_1_^+^|-field and SAR map were normalized using the calculated normalization factors as shown in Figure 4. The normalization factors were separately compared for three cases and showed different characteristics for each case. The normalization factors were compared with regard to three cases: (i) a case in which the diameter increased from 300 to 500 mm; (ii) a case in which the length increased from 210 to 350 mm; and (iii) a case in which both the diameter and length increased while maintaining a D/L-ratio of 0.7. With the exception of the 11.7 T results obtained when using the cylindrical phantom model, the normalization factors exhibited decreases with an increasing coil diameter and increases with an increasing coil length. On the other hand, a notable tendency was identified when the D/L-ratio was fixed at 0.7 and the coil diameter and length increased simultaneously. The cylindrical phantom model and human body model results demonstrated different tendencies when the D/L-ratio was fixed at 0.7 and the diameter and length increased simultaneously. At 3.0 T, the cylindrical phantom model results showed linear increases in their normalization factors as the diameter and length increased simultaneously and nonlinear increases at 7.0 and 11.7 T. On the other hand, the human body model results at 3.0 and 7.0 T showed linear increases in its normalization factors, but at 11.7 T, the normalization factors increased rapidly when moving from a 300 mm diameter and 210 mm coil length to a 350 mm diameter and 245 mm coil length and decreased rapidly in the case of a 400 mm diameter and 280 mm coil length. From an RF power perspective, these results are expected to enable an effective RF transmission if the coil diameter is as close to the object as possible or if the coil is large enough in terms of both its diameter and its length.

**Figure 5:**
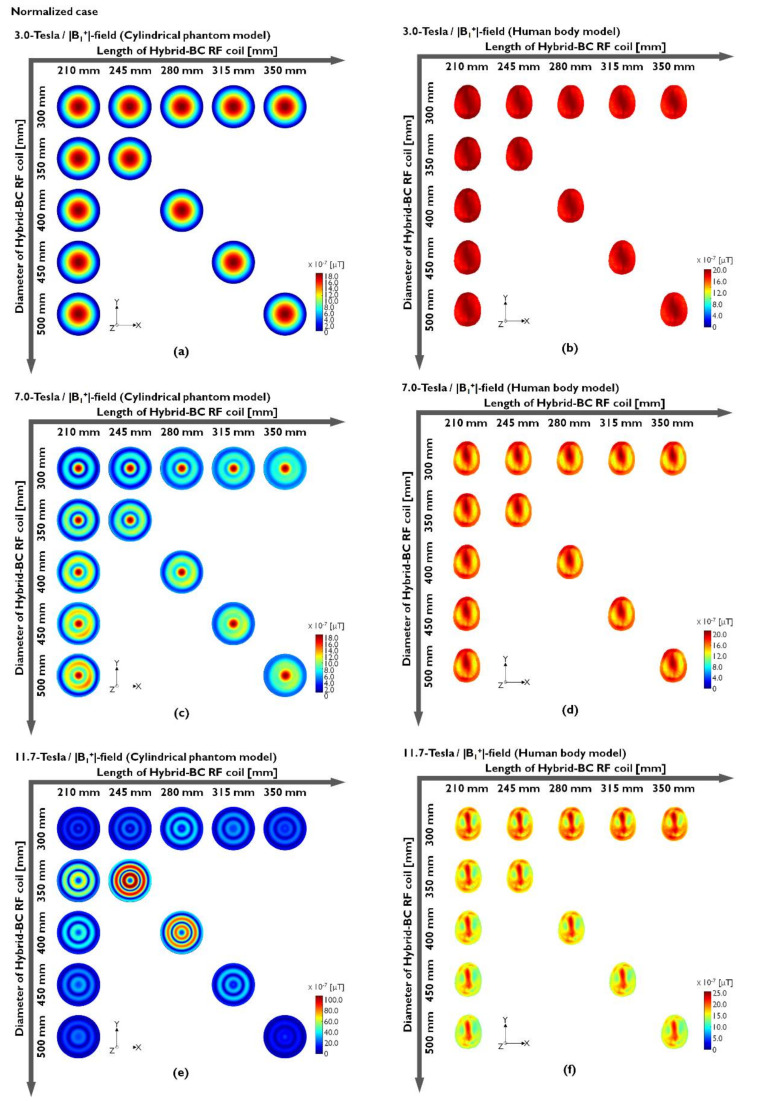
Normalized |B_1_^+^|-field distribution obtained when using a cylindrical phantom model (**a**,**c**,**e**) or a human body model (**b**,**d**,**f**).

To obtain normalized |B_1_^+^|-field distributions, the normalization factors of the normalized |B_1_^+^|-fields were applied to the unnormalized |B_1_^+^|-field maps of the cylindrical phantom model (using distilled water) and of the human body model, as shown in Figure 5 and Table 2. In Figure 5a, the cylindrical phantom model results at 3.0 T revealed slightly inhomogeneous normalized |B_1_^+^|-field maps that can be validated because of their central brightness effect because of their RF reduction. On the other hand, in Figure 5b, the results of the human body model indicate a more uniform normalized |B_1_^+^|-field as compared to the cylindrical phantom model results. In the 7.0 T simulation results using the cylindrical phantom model (Figure 5c), the normalized |B_1_^+^|-field presented with nonuniformity due to the ring-shaped field distributions and the fact that the RF fields were still concentrated at the center of the normalized |B_1_^+^|-field. However, the human model results at 7.0 T provided a satisfactory normalized |B_1_^+^|-field distribution, except for the normalized |B_1_^+^|-field focusing on the center of the human body model (Figure 5d). The cylindrical phantom model results at 11.7 T (Figure 5e) demonstrated a normalized |B_1_^+^|-field distribution of the double ring shapes without the central concentration of the cylindrical phantom model. However, the simulation results of the human body model tended to be analogous to those of the cylindrical phantom model results at 7.0 T and confirmed that the normalized |B_1_^+^|-field uniformity improves as the length of the Hybrid-BC RF coil increases (Figure 5f).

**Figure 6:**
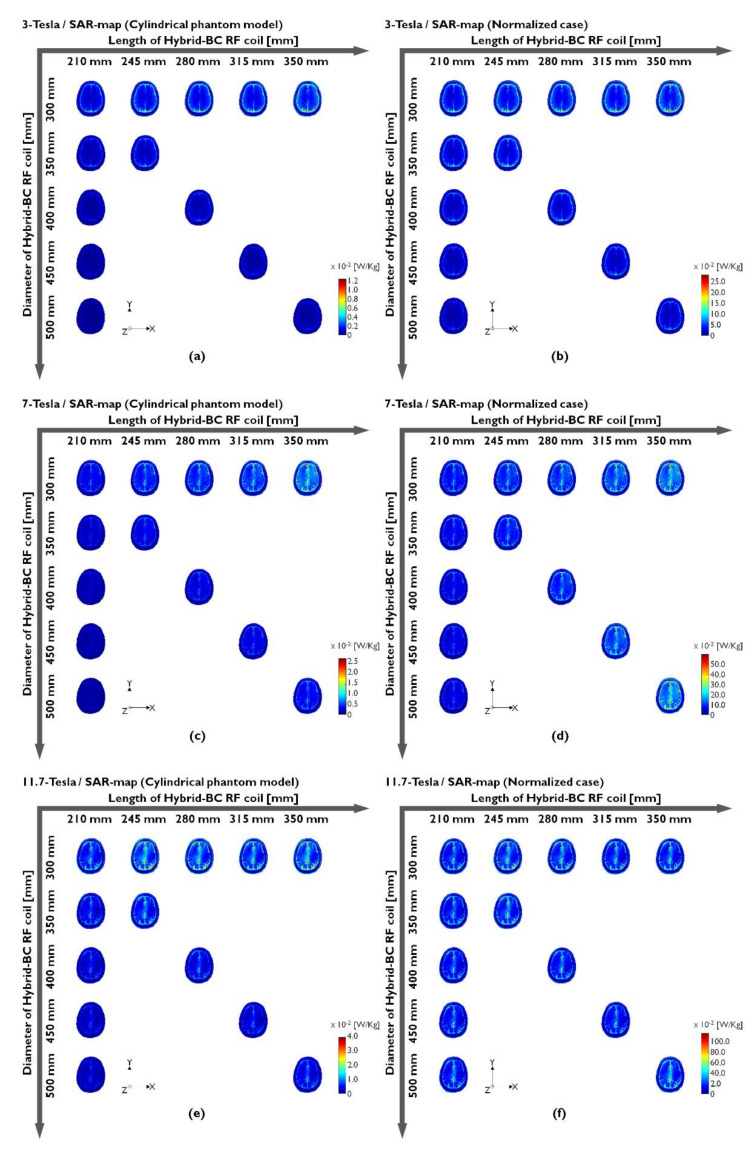
SAR maps generated under unnormalized (**a**,**c**,**e**) or normalized (**b**,**d**,**f**) conditions when using a human body model.

The SAR maps were compared with the unnormalized (Figure 6a,c,e) and normalized (Figure 6b,d,f) SAR maps. In the EM-field simulation of the RF coils for MRI, the local SAR values were calculated using the |E|-fields (Figure S5 and Table S3), which were generated when the current drives through the RF coil without specifying the strength of the applied RF signal. The unnormalized SAR map was computed as a ratio between the dissipated power and its mass densities. This can be indicated in other expressions as local or unaveraged SAR. On the other hand, the normalized SAR map was verified by applying the calculated normalization factors (shown in Figure 5) to the unnormalized SAR map. As shown in the unnormalized SAR maps in the left column of Figure 6, the SAR distribution increases with the length of the Hybrid-BC RF coil. However, an increase in the SAR distribution was not seen when the diameter and length of the RF coil increased simultaneously (as shown in Figure 6a,c,e). Moreover, the quantification of the unnormalized SAR maps was difficult because the conditions under which the RF power was applied were not taken into account when applying the π/2 RF pulses on the chosen methods for the computation of the existing SAR maps. However, for the normalized SAR maps in the right column of Figure 6, a quantitative analysis was possible by assuming the applied π/2 RF pulse-induced conditions. The normalized SAR map results (Figure 6b,d,f) confirmed that—unlike the unnormalized SAR map results—the SAR distribution increased along with the increasing coil diameter and length. As the diameter and length of the Hybrid-BC RF coil increased, the RF power of the π/2 RF pulse required to excite the proton in the object also increased, thus increasing the real SAR distribution. In the 3.0 T normalized SAR map results presented in Figure 6a, the normalized SAR distribution was concentrated at the periphery of the human brain. The normalized SAR map results at 7.0 T (Figure 6d) showed that the SAR distribution tends to focus on the center of the brain, eventually confirming that it is extremely concentrated in the same area at 11.7 T (Figure 6f). Table 2 presents the STD values of the normalized |B_1_^+^|-field along with the normalization factor values and Table 3 presents peak SAR values of the unnormalized and normalized SAR maps.

In summary, the uniformity evaluation results obtained using the STD values of the normalized |B_1_^+^|-field revealed that the size of the Hybrid-BC RF coil with the maximum uniformity tended to vary depending on the type of objects used and generated B_0_-field strength. In both the cylindrical phantom model and human body model results, the uniformity of the normalized |B_1_^+^|-field linearly increased as the coil length increased, and it decreased as the coil diameter increased at 3.0 T. In cases in which the coil diameter and length increased at the same time, the normalized |B_1_^+^|-field uniformity was found to decrease linearly at the cylindrical phantom model results, and at the human body model results, it was shown to demonstrate V-shaped uniformity changes. In fact, uniformity was shown to decrease from a coil D/L-ratio of 300/210 to a coil D/L-ratio of 400/280 and to subsequently increase from a D/L-ratio of 400/280 to a D/L-ratio of 500/350. At 7.0 T, when using the cylindrical phantom model, the V-shaped uniformity changes were also observed when the coil diameter increased while the length remained fixed at 210 mm. In cases in which the coil length increased and the diameter remained fixed at 300 mm, the STD values linearly decreased up until a coil length of 315 mm and then increased again at a length of 350 mm. In cases in which the coil diameter and length increased simultaneously, the STD values demonstrated a linear decrease of their distributions, with slight changes. In that respect, the human body model results tended to be analogous to those of the cylindrical phantom model results at 3.0 T, and the linear increments in the recorded STD values were maintained as the coil diameter and length increased. In the cylindrical phantom model results obtained at 11.7 T, the rapidly changing STD values were observed at a coil diameter of 350 mm with a length of 210 mm, and the STD distribution of an inverted V-shape (hat-shape) could be identified.

These hat-shaped STD distributions represent the limits of the Hybrid-BC RF coil size. The points representing the lowest or highest STD values (in the V-shaped or the hat-shaped distributions) help us determine the optimal size of the Hybrid-BC RF coil. In cases in which both the coil diameter and length increased simultaneously while maintaining a fixed D/L-ratio of 0.7, the STD value increased rapidly by 5.5 times from a coil diameter of 350 mm and length of 245 mm to a coil diameter of 300 mm and length of 200 mm. Owing to the normalized |B_1_^+^|-field inhomogeneity caused by the RF shortening and dielectric properties of the cylindrical phantom model (using distilled water), the performance of the Hybrid-BC RF coil could not be evaluated using the cylindrical phantom model at 11.7 T. On the other hand, the human body model results obtained at 11.7 T revealed the analogousness of their STD distributions similar to that of the cylindrical phantom model results at 7.0 T. The minimum STD value was measured at a coil diameter of 350 mm and length of 245 mm. In terms of the normalized |B_1_^+^|-field uniformity, the Hybrid-BC RF coil specifications optimized for the B_0_-field strength must be used. At the 3.0 T MRI system, the optimal size of the Hybrid-BC RF coil was suggested to bear a diameter of 400 mm and length of 280 mm. At the 7.0 T MRI system, the optimal coil dimensions were recommended to be those of a 500 mm diameter and 350 mm length. The optimal reference RF coil size for the newly installed 11.7 T MRI system at the GUGMC is recommended to bear a diameter of 350 mm and length of 245 mm.

The SAR prediction method was proposed for the undertaking of the RF safety evaluations in the case of ultrahigh-field MRI, and the method was performed by applying a normalized SAR technique to more quantitatively predict the SAR values. Normalized peak SAR values tended to decrease as the coil diameter increased and to increase as the coil length increased at 3.0 T. The normalized peak SAR values decreased by approximately 36% as the coil dimensions increased from a D/L-ratio of 300/210 (0.2025 W/kg) to a D/L-ratio of 500/350 (0.1284 W/kg), with a D/L-ratio of 0.7. This is because the normalized peak SAR value is a sensitive factor due to the increase of the Hybrid-BC RF coil diameter. On the other hand, the 7.0 T results were influenced by increased coil diameter and length, and the normalized peak SAR increased by 74% when the dimensions of the coil increased from a D/L-ratio of 300/210 (0.3408 W/kg) to a D/L-ratio of 500/350 (0.5952 W/kg) under a fixed D/L-ratio at 0.7. With regard to the 11.7 T system, the difference in the observed normalized peak SAR value was negligible and within a 10% range when the dimensions of the Hybrid-BC RF coil increased from a D/L-ratio of 300/210 (0.8215 W/kg) to a D/L-ratio of 450/315 (0.8158 W/kg) under a fixed D/L-ratio at 0.7, but it increased by approximately 40% for a coil D/L-ratio dimension of 500/350 (1.1551 W/kg).

In terms of the SAR values obtained at 3.0 T, the optimal size of the Hybrid-BC RF coil was recommended to be one of a diameter of 500 mm and length of 210 mm by its ability to exert the minimum normalized peak SAR value. At 7.0 T, the Hybrid-BC RF coil with a diameter of 450 mm and length of 210 mm exerted a minimum normalized peak SAR value; this same structure was recommended as an optimal size of a reference RF coil at 7.0 T. The optimal reference RF coil dimensions for the 11.7 T system (that exists in the GUGMC) are suggested to be one of a diameter of 350 mm and length of 210 mm. The SAR values decreased with the increase of the coil diameter and increased with the increase of the coil length. On the basis of these trends, it is advantageous in terms of SAR to minimize the length of the reference RF coil and to enlarge its diameter, but the uniformity of the generated normalized |B_1_^+^|-fields should also be taken into account to determine the size of the reference RF coil based on the available SAR value.

In this work, EM-field simulation was performed to propose an optimized diameter and length of the BC RF coil for its use as a reference coil in the 11.7 T MRI system being newly installed at the GUGMC. The EM-field simulation results for 11.7 T were compared with those for 3.0 and 7.0 T to propose an optimized RF coil for different B_0_-field strengths. The BC RF coil is usually chosen for MRI because of its ability to achieve circular polarization with high homogeneity under symmetric conditions. However, owing to the increase in the generated B_0_-field strength, there is a problem with the nonuniformity of the |B_1_|-fields due to RF reduction. Despite |B_1_|-field inhomogeneity, the BC RF coil is mostly used as a reference RF coil for the assessment of the performance of MRI systems as well as for the reduction of the mutual inductance or magnetic coupling between the coil and other electronics during practical MR imaging. For this reason, the BC coil was selected as a reference coil, and EM-field simulation was performed using a Hybrid-BC RF coil with the least change in its signal sensitivity as indicated by the B_0_-field strength and highest signal sensitivity at UHF.

In EM-field simulation, we compared the cylindrical phantom models and human models with regard to changes in the B_0_-field strength. The cylindrical phantom model’s results obtained at 3.0 T showed an EM-field distribution analogous to that of the human body model results obtained at 7.0 T. Furthermore, analogousness was shown in the 7.0 T cylindrical phantom model’s results as well as in the 11.7 T human body model’s results. From the EM-field simulation results, the effect of changes in coil diameter and length of the Hybrid-BC RF coil on EM-field distribution has been identified according to the B_0_-field strength. As the diameter improved while the length of the Hybrid-BC RF coil was fixed, the normalization factors of the normalized |B_1_^+^|-field increased. Conversely, the RF transmission efficiency decreased as the diameter was fixed and the length increased. In terms of EM-field sensitivity and uniformity, the |B_1_^+^|-field was associated with the sensitivity of the |B_1_|-field and the phase map of the |B_1_^−^|-field has been found to be related to the uniformity of the |B_1_|-field. As shown by the use of the cylindrical phantom model’s results at 11.7 T, the uniformity when the coil diameter and length increase at the same time with a fixed D/L-ratio of 0.7 is almost unpredictable.

From the obtained EM-field simulation results, we investigated the optimal diameter and length for the Hybrid-BC RF coils by interrogating three factors: the unnormalized |B_1_^+^|-field sensitivity, normalized |B_1_^+^|-field uniformity, and normalized SAR. In particular, we analyzed the normalization factor in terms of its |B_1_^+^|-field sensitivity, the normalized |B_1_^+^|-field in terms of its |B_1_^+^|-field uniformity, and the normalized SAR in terms of its RF safety. The order in which the optimized coil diameter and length were defined is as follows. First, we selected the Hybrid-BC RF coil with the diameter and length that provide the maximum |B_1_^+^|-field sensitivity in terms of normalization factors and compared the generated STD values to determine its suitability in terms of normalized |B_1_^+^|-field uniformity. Finally, RF safety was verified adaptability in terms of SAR to determine human safety.

Among the three evaluation factors for the optimal diameter and length of Hybrid-BC RF coil, the result to be noted in this study is the normalization factor that can be defined as the RF transmission efficiency of the Hybrid-BC RF coil. The normalization factor of the unnormalized |B_1_^+^|-field indicates the signal sensitivity of the normalized |B_1_^+^|-field. In the normalization factor results summarized in Table 2, both the cylindrical phantom model and human body model results revealed that the minimum values of the normalization factor were measured in the following order of coil D/L-ratio: 300/350 (1st), 300/315 (2nd), and 300/280 (3rd) at 3.0 and 7.0 T. At 11.7 T, the normalization factor showed slightly different results between the cylinder phantom and human body model. In the cylindrical phantom model results, the minimum normalization factor values were identified in the following order of coil D/L-ratio: 300/245 (1st), 300/210 (2nd), and 300/350 (3rd). In human body model results, the minimum normalization factor values were identified in the following order of coil D/L-ratio: 300/245 (1st), 300/280 (2nd), and 300/350 (3rd).

In Figure 4, the tendency of variation in the normalization factors differed depending on the B_0_-field strength and the types of numerical phantom models. To analyze the tendency of variation in the normalization factor, RF energy efficiency of Hybrid-BC coil were analyzed as a percentage with the difference between the lowest normalization value and largest normalization value of Hybrid-BC RF coil in Table 4. In terms of RF transmission efficiency, an increase in the normalization factor means a decrease in RF transmission efficiency, and a decrease in the normalization factor means an increase in RF transmission efficiency.

In the 3.0 T results, the RF transmission efficiency according to changes in the length and diameter of Hybrid-BC coil showed similar tendencies in the cylinder phantom model and human body model. In addition, a linear decrease in RF transmission efficiency was observed when the diameter and length were increased at a fixed D/L-ratio of 0.7. On the other hand, the results of 7.0 T and 11.7 T results showed different variations in RF transmission efficiency depending on the types of numerical phantom models.

In particular, a case in which both the diameter and length increased while maintaining a D/L-ratio of 0.7 in the 7.0 T results, the normalization factors provided almost constant RF transmission efficiency without linear increase or decrease. This result shows that the B_1_^+^-field transmission efficiency in UHF MRI was determined not by the structure of the RF coil, but by the dielectric properties and geometry of loaded object [68,69].

In the 11.7 T result, the inhomogeneity in the B_1_^+^-field becomes severe due to the RF wavelength being 1.67 times shorter than the 7.0 T [70,71,72,73,74]. For this reason, it was difficult to find regularity for RF transmission efficiency changes due to the increase in the diameter and length (D/L-ratio fixed 0.7) of the hybrid-BC RF coil.

In terms of the normalized |B_1_^+^|-field uniformity and normalized SAR values for each of the B_0_-field strengths, we performed a conformity assessment for the three sizes of the Hybrid-BC RF coils with the maximum |B_1_^+^|-field sensitivity as determined using the normalization factor results. We were able to confirm that the STD values of the normalized |B_1_^+^|-field and SAR values for the three sizes of the Hybrid-BC RF coils with the maximum unnormalized |B_1_^+^|-field sensitivity were within an acceptable range.

In fact, the minimum STD values of the normalized |B_1_^+^|-field were observed depending on the B_0_-field strength and types of the numerical phantoms. In the results obtained by the testing of the cylindrical phantom model, the specifications of the Hybrid-BC RF coil depending on the B_0_-field strength were determined to be those of a D/L-ratio of 300/350 at 3.0 T, a D/L-ratio of 350/245 at 7.0 T, and a D/L-ratio of 500/350 at 11.7 T. Meanwhile, in the human body phantom results, the optimal specifications of the Hybrid-BC RF coil were decided to be those of a D/L-ratio of 500/210 at 3.0 T, a D/L-ratio of 500/210 at 7.0 T, and a D/L-ratio of 350/245 at 11.7 T. For the normalized SAR values, the dimensions of the Hybrid-BC RF coil that exert the minimum SAR value were decided to be those of a D/L-ratio of 500/210 at 3.0 T, a D/L-ratio of 450/210 at 7.0 T, and a D/L-ratio of 350/210 at 11.7 T.

However, the optimal dimensions of the Hybrid-BC RF coil that were investigated in terms of their minimum-produced STD and SAR values have a problem of bearing relatively large diameters (almost at the level of body coil sizes) and of requiring more RF power as a result of not considering the generated |B_1_^+^|-field sensitivity. For these reasons, we assessed the optimal size of the Hybrid-BC RF coil in terms of its generated |B_1_^+^|-field sensitivity, and we considered only a specific range of conformity within a certain range of STD values and SARs.

In addition, this study was performed to determine the specification of the reference RF coil on a newly installed 11.7 T MRI system. However, our study has two limitations. First, the maximum diameter of the Hybrid-BC coil was 500 mm, but when MRI was used to acquire patient images, only the head image was not acquired using the Hybrid-BC RF coil. For the systems currently installed at the GUGMC, the patient aperture is 570 mm, thus allowing for the use of body coils of up to 530 mm in diameter. However, EM-field simulations were performed for up to 500 mm in coil diameter to limit the results. On the other hand, in experiments with reference RF coils, EM-field simulations were performed with a sufficient diameter and length to allow the performance evaluation of MRI systems, with the exception of body coils.

Second, the proposed SAR calculation method of using the local SAR along with normalization factors was analyzed in detail. The proposed SAR calculation method produced a more quantitative SAR analysis that is analogous to the MRI experiments performed on Spin-echo RF pulse sequences. The proposed method can help with the acquisition of more quantitative local SAR prediction. Hence, the proposed SAR calculation method is too sensitive because the local SAR is too sensitive to approximation because of the computational methods employed. Furthermore, the energy deposited at a certain point is invariably smeared out because of heat conduction. Therefore, single point values might not be thermally significant. For these reasons, the SAR is mostly presented in an averaged form. For future studies, the proposed SAR calculation method using the normalization factor should be conducted by applying the mean SAR values (such as 1 and 10 g mean SARs) to perform more accurate SAR analyses.

Research on the development of extremely ultrahigh-field (EUHF) MRI above 11.7 T has started worldwide [75]. The increased intensity of magnetic field will allow the acquisition of anatomical and functional information at high resolution. Among the various practical designs of RF coils used for NMR imaging and EUHF MRI, birdcage RF coils are widely used, with applications ranging from low-field MRI systems to ultra-high-end MRI systems [76,77,78,79,80,81,82,83,84,85,86,87,88,89,90]. Recently, BC RF coils have been actively used in clinical applications [8,83,84] and pre-clinical animal experimental studies [85,86]. In particular, it is widely used in multinuclear MRI requiring high SNR and uniformity [77,87,88,89,90]. The optimized Hybrid-BC RF coil size proposed in this paper can be applied to various B_0_-field strengths (3.0 T, 7.0 T and 11.7 T) to obtain MR images that provide higher SNR, uniformity and RF safety in clinical applications and preclinical studies. In particular, it is expected that low B_1_^+^-field sensitivity can be improved by applying optimal size of Hybrid BC RF coil to multinuclear MRI, which has relatively low B_1_^+^-field sensitivity compared to proton MRI. This paper evaluated Hybrid-BC RF coils with sizes optimized for HF, UHF, and EUHF MRI. For increasing the strength of the B_0_-field, further research is being conducted to optimize the size of BC coils. If RF coils are used considering the limitations of individual MRI systems, MR images with high B_1_^+^-field sensitivity and uniformity can be obtained at the desired B_0_-field strength.

## 4. Conclusions

The suitability of Hybrid-BC RF coils for use as reference RF coils in UHF MRI systems was analyzed and evaluated with regard to three aspects: their EM-field sensitivity, their EM-field uniformity, and their RF safety. To evaluate their EM-field sensitivity, we analyzed the magnitude maps (|B_1_|-, |B_1_^+^|-, and |B_1_^−^|-fields) under the unnormalized conditions as well as the normalization factors of the unnormalized |B_1_^+^|-field. In terms of EM-field uniformity, we evaluated the obtained phase maps (phase maps of the B_1_-, B_1_^+^-, and B_1_^−^-fields) under the unnormalized conditions as well as the STD values of the normalized |B_1_^+^|-field. Finally, for RF safety, the SAR maps were validated under the normalized conditions by applying the normalization factor to the unnormalized SAR values.

As shown by the obtained EM-field simulation results under the unnormalized conditions, the increase of the RF coil diameter causes a reduction of the |B_1_^+^|-field sensitivity, thus requiring more RF power. We also confirmed that EM-field inhomogeneity was caused by an increase in the B_0_-field strength and that the latter was accompanied by a distortion of RF transmission and reception. Under the normalized conditions and regarding |B_1_^+^|-field uniformity, dramatic changes in the STD values could not be observed with changes in the diameter and length of the simulated Hybrid-BC RF coil. With regard to the normalized SAR, it has been found that coils of a certain diameter and length exhibit better SAR distribution for each of the tested B_0_-field strengths.

In this work, we identified the diameter and length of the Hybrid-BC RF coil that provided optimal performance in each of the tested B_0_-field strengths, and as a result, we defined the size of the Hybrid-BC RF coil that is suitable for use as a reference coil. The optimal size of the Hybrid-BC RF coil was proposed based on the |B_1_^+^|-field sensitivity, and only a conformity assessment was considered in terms of the STD values and SARs. The coil dimensions are suggested to be a D/L-ratio of 300/350 at 3.0 and 7.0 T and a D/L-ratio of 300/245 at 11.7 T.

Based on the optimal diameter and length proposed in this paper, we are planning to apply the Hybrid-BC RF coil with a diameter of 300 mm and length of 245 mm as the reference RF coil in the 11.7 T MRI system currently being developed at the GUGMC. Furthermore, we expect that the optimal size of the Hybrid-BC RF coil herein proposed as a reference coil will be effectively applied to other UHF MRI systems. Further research on the optimized D/L-ratio and types of BC coils from the perspective of the B_1_^+^-field is required to solve the inhomogeneity of the B_1_-field arising due to the increase in the B_0_-field identified in this study.

## Figures and Tables

**Table 1:** Unnormalized |B_1_^+^|-field values obtained when using a cylindrical phantom model or a human body model at a 3.0, 7.0, or 11.7 T MRI system.

**Cylindrical Phantom Model (Distilled Water)**											
	**L (mm)**	**Center-Point Values [×10^−6^ μT]**					**Mean Values [×10^−6^ μT]**				
**D (mm)**		**210**	**245**	**280**	**315**	**350**	**210**	**245**	**280**	**315**	**350**
3.0 T	300	0.681	0.827	0.903	0.956	0.106	0.269	0.334	0.371	0.397	0.444
	350	0.507	0.625				0.201	0.252			
	400	0.384		0.518			0.153		0.212		
	450	0.300			0.429		0.120			0.177	
	500	0.239				0.400	0.096				0.165
7.0 T	300	0.248	0.273	0.299	0.328	0.373	0.061	0.074	0.092	0.111	0.134
	350	0.142	0.167				0.045	0.056			
	400	0.100		0.158			0.038		0.058		
	450	0.082			0.176		0.033			0.067	
	500	0.071				0.205	0.029				0.081
11.7 T	300	0.551	0.556	0.357	0.422	0.465	0.280	0.365	0.359	0.349	0.310
	350	0.175	0.124				0.289	0.331			
	400	0.218		0.102			0.250		0.232		
	450	0.259			0.165		0.204			0.163	
	500	0.292				0.339	0.171				0.146
**Human Body Model (Duke Phantom)**											
	**L (mm)**	**Center-Point Values [×10^−6^ μT]**					**Mean Values [×10^−6^ μT]**				
**D (mm)**		**210**	**245**	**280**	**315**	**350**	**210**	**245**	**280**	**315**	**350**
3.0 T	300	0.071	0.082	0.083	0.083	0.093	0.069	0.077	0.077	0.077	0.086
	350	0.054	0.064				0.053	0.060			
	400	0.043		0.053			0.041		0.049		
	450	0.034			0.044		0.033			0.041	
	500	0.028				0.042	0.026				0.039
7.0 T	300	0.060	0.070	0.075	0.080	0.092	0.051	0.058	0.062	0.066	0.076
	350	0.047	0.057				0.040	0.047			
	400	0.039		0.055			0.033		0.046		
	450	0.033			0.057		0.028			0.047	
	500	0.030				0.063	0.025				0.053
11.7 T	300	0.079	0.093	0.088	0.083	0.088	0.070	0.084	0.081	0.077	0.083
	350	0.070	0.081				0.059	0.070			
	400	0.061		0.069			0.050		0.058		
	450	0.056			0.063		0.044			0.051	
	500	0.053				0.072	0.041				0.057

**Table 2:** STD values of the normalized |B_1_^+^|-field with normalization factors at a 3.0, 7.0, or 11.7 T MRI system.

**Cylindrical Phantom Model (Distilled Water)**											
	**L (mm)**	**Normalization Factors [a.u.]**					**STD of Normalized |B_1_^+^|-field [× 10^−6^ μT]**				
**D (mm)**		**210**	**245**	**280**	**315**	**350**	**210**	**245**	**280**	**315**	**350**
3.0 T	300	2.876	2.368	2.168	2.048	1.846	0.215	0.210	0.207	0.204	0.202
	350	3.861	3.132				0.215	0.211			
	400	5.101		3.776			0.214		0.208		
	450	6.539			4.562		0.213			0.206	
	500	8.195				4.897	0.211				0.204
7.0 T	300	7.894	7.168	6.543	5.974	5.239	0.138	0.131	0.126	0.122	0.131
	350	13.812	11.722				0.121	0.116			
	400	19.572		12.360			0.118		0.118		
	450	23.983			11.108		0.120			0.128	
	500	27.488				9.541	0.138				0.136
11.7 T	300	3.552	3.518	5.486	4.634	4.214	0.187	0.246	0.373	0.306	0.241
	350	11.193	15.750				0.645	1.042			
	400	8.982		19.221			0.448		0.866		
	450	7.552			11.861		0.312			0.354	
	500	6.692				5.777	0.237				0.143
**Human Body Model (Duke Phantom)**											
	**L (mm)**	**Normalization Factors [a.u.]**					**STD of Normalized |B_1_^+^|-field [× 10^−6^ μT]**				
**D (mm)**		**210**	**245**	**280**	**315**	**350**	**210**	**245**	**280**	**315**	**350**
3.0 T	300	27.556	24.006	23.683	23.597	21.015	0.018	0.015	0.016	0.017	0.019
	350	35.973	30.773				0.017	0.014			
	400	45.852		37.297			0.016		0.014		
	450	57.364			44.273		0.015			0.016	
	500	70.429				46.176	0.013				0.017
7.0 T	300	32.437	27.853	26.087	24.407	21.199	0.056	0.060	0.062	0.063	0.060
	350	41.781	34.551				0.059	0.058			
	400	50.575		35.516			0.057		0.057		
	450	58.789			34.435		0.056			0.057	
	500	65.341				31.205	0.054				0.055
11.7 T	300	24.685	21.103	22.141	23.708	22.149	0.067	0.066	0.066	0.065	0.062
	350	28.169	24.172				0.063	0.061			
	400	31.964		28.335			0.067		0.063		
	450	35.028			31.294		0.069			0.066	
	500	36.804				27.399	0.071				0.066

**Table 3:** Peak SAR values under unnormalized and normalized conditions at a 3.0, 7.0, or 11.7 T MRI system.

**Human Body Model (Duke Phantom)**											
	**L (mm)**	**Unnormalized Peak SAR [W/kg]**					**Normalized Peak SAR [W/kg]**				
**D (mm)**		**210**	**245**	**280**	**315**	**350**	**210**	**245**	**280**	**315**	**350**
3.0 T	300	0.007	0.010	0.010	0.011	0.013	0.203	0.239	0.240	0.248	0.280
	350	0.005	0.007				0.183	0.217			
	400	0.003		0.006			0.143		0.204		
	450	0.002			0.004		0.117			0.169	
	500	0.001				0.003	0.081				0.128
7.0 T	300	0.012	0.017	0.019	0.021	0.028	0.341	0.398	0.446	0.492	0.590
	350	0.007	0.010				0.247	0.317			
	400	0.006		0.011			0.251		0.400		
	450	0.004			0.010		0.199			0.454	
	500	0.003				0.013	0.207				0.595
11.7 T	300	0.030	0.040	0.038	0.035	0.042	0.822	0.967	0.908	0.816	0.879
	350	0.020	0.031				0.714	0.943			
	400	0.016		0.022			0.752		0.821		
	450	0.013			0.018		0.732			0.816	
	500	0.013				0.025	0.914				1.155

**Table 4:** The variations of RF transmission efficiency according to the change in diameter or length of Hybrid-BC RF coil in a 3.0, 7.0, or 11.7 TRI system.

B_0_-Field	Numerical Phantom	Length Increase	Diameter Increase	Diameter and Length Increase
3.0 T	Cylindrical phantom model	35.813%	−184.944%	−70.2712%
	Human body model	23.737%	−155.585%	−67.5715%
7.0 T	Cylindrical phantom model	33.633%	−248.214%	−56.574%
	Human body model	34.645%	−101.44%	−9.492
11.7 T	Cylindrical phantom model	−54.448%	−215.118%	−441.132
	Human body model	14.510%	−49.0946%	−26.773

## Data Availability

Not applicable.

## Supplementary Materials

The following supporting information can be downloaded at: https://www.mdpi.com/article/10.3390/s22041512/s1: Figure S1. Unnormalized |B_1_^−^|-field distribution obtained when using a cylindrical phantom model (a,c,e) or a human body model (b,d,f); Figure S2. Phase map of the unnormalized B_1_^−^-field distribution obtained when using a cylindrical phantom model (a,c,e) or a human body model (b,d,f); Figure S3. Unnormalized |B_1_|-field distribution obtained when using a cylindrical phantom model (a,c,e) or a human body model (b,d,f); Figure S4. Phase map of the unnormalized B_1_-field distribution obtained when using a cylindrical phantom model (a,c,e) or a human body model (b,d,f); Figure S5. Unnormalized |E|-field distribution obtained when using a cylindrical phantom model (a,c,e) or a human body model (b,d,f); Table S1. Unnormalized |B_1_^−^|-field values obtained when using a cylindrical phantom model or a human body model at a 3.0, 7.0, or 11.7 T MRI system; Table S2. Unnormalized |B_1_|-field values obtained when using a cylindrical phantom model or a human body model at a 3.0, 7.0, or 11.7 T MRI system; Table S3. Unnormalized |E|-field values obtained when using a cylindrical phantom model or a human body model at a 3.0, 7.0, or 11.7 T MRI system.

## List of Symbols

The following symbols are used in this manuscript:

λ	Larmor frequencies
ε_r_	Relative permittivity
σ	Conductivity
$B_{1x}$	x-component of magnetic flux density field
$B_{1y}$	y-component of magnetic flux density field
$B_{1z}$	z-component of magnetic flux density field
$B_{1xy}$	Transverse component of magnetic flux density field
$B_{1\_phase}$	Phase of magnetic flux density field
abs	Absolute value
L	Power loss
σΕ	Conduction current
Ε	Time-harmonic electric field phasor
${Ε}^{*}$	Conjugate transpose of time-harmonic electric field phasor
r	Locations of calculation
ρ	Mass density
T	Temperature

## Abbreviations

MRI	Magnetic resonance imaging
T	Tesla (A derived unit of the magnetic B-field strength)
RF	Radiofrequency
B_0_-field	Magnetic field
B_1_-field	Magnetic flux density field
|B_1_|-field	Magnitude map of magnetic flux density field
B_1_^+^-field	RF transmission field
|B_1_^+^|-field	Magnitude map of RF transmission field
B_1_^-^-field	RF reception field
|B_1_^-^|-field	Magnitude map of RF reception field
E-field	Electric field
|E|-field	Magnitude map of electric field
BC	Birdcage coil
LP-BC	Low-pass-type birdcage coil
HP-BC	High-pass-type birdcage coil
Hybrid-BC	Hybrid-type birdcage coil (Band-pass-type birdcage coil)
LF	Low-field
HF	High-field
UHF	Ultra-high-field
EUHF	Extremely-ultra-high-field
SAR	Specific absorption rate
D	Diameter
L	Length
D/L-ratio	Diameter-to-length ratio
EM-field	Electromagnetic field
CP	left-point
STD	Standard deviation
GUGMC	Gachon University Gil medical left
FDTD	Finite-difference time-domain
IT’IS	The foundation for research on information technologies in society
GPU	Graphics processing unit
CPU	Central processing unit
CUDA	Compute unified device architecture
