# Supplementary material for: A Preliminary Study for Reference RF Coil at 11.7 T MRI: Based on Electromagnetic Field Simulation of Hybrid-BC RF Coil According to Diameter and Length at 3.0, 7.0 and 11.7 T
Source: Sensors (Basel). 2022 Feb 15;22(4):1512. doi: 10.3390/s22041512 (PMC8875900; doi:10.3390/s22041512)
Supplement: Supplementary file 1 [file sensors-22-01512-s001.zip › sensors-1573789-supplementary.pdf]

sensors

MDPI

Supplementary Materials

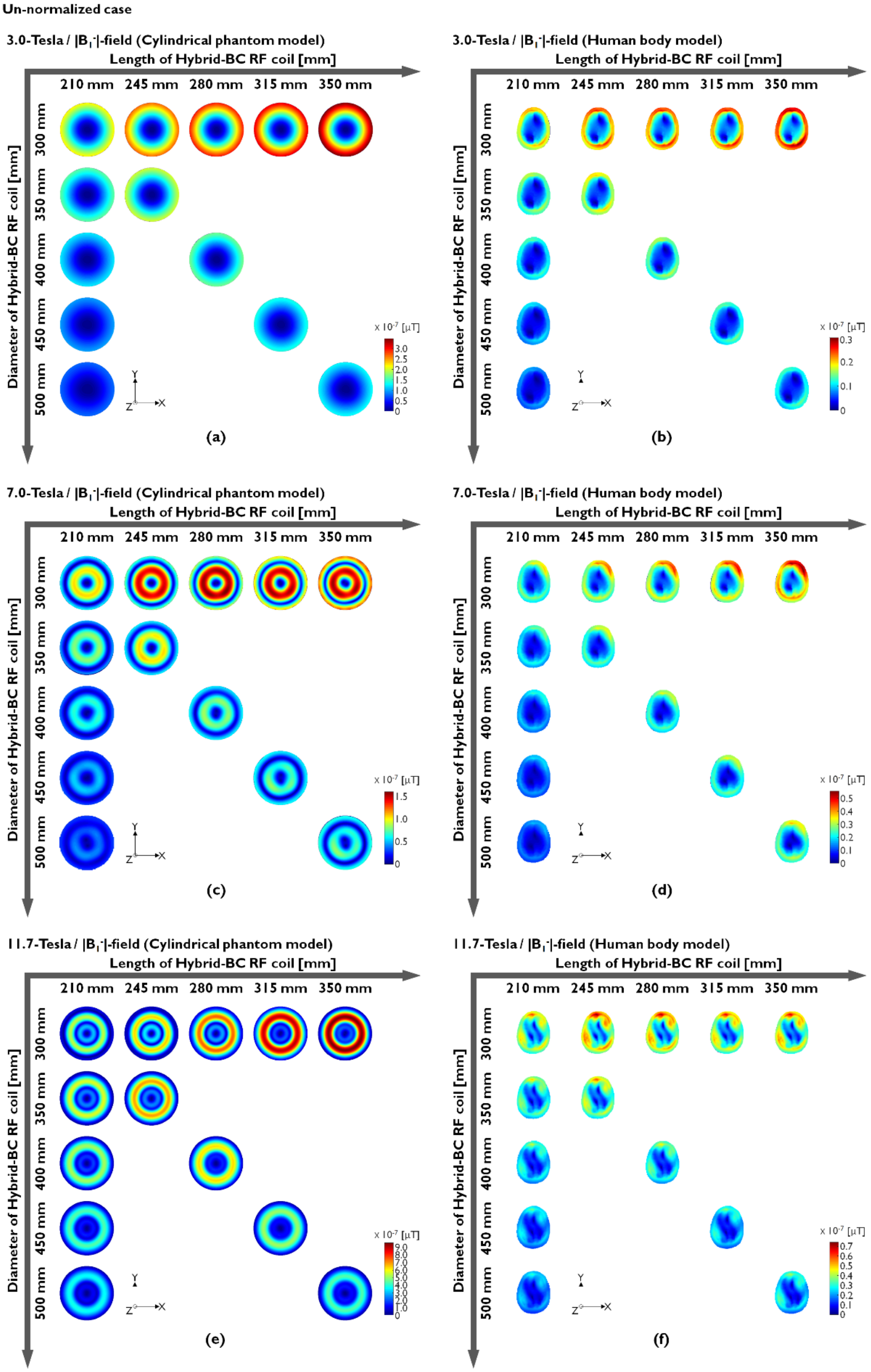

Figure S1. Unnormalized $|B_1^-|$-field distribution obtained when using a cylindrical phantom model (**a**, **c**, and **e**) or a human body model (**b**, **d**, and **f**).

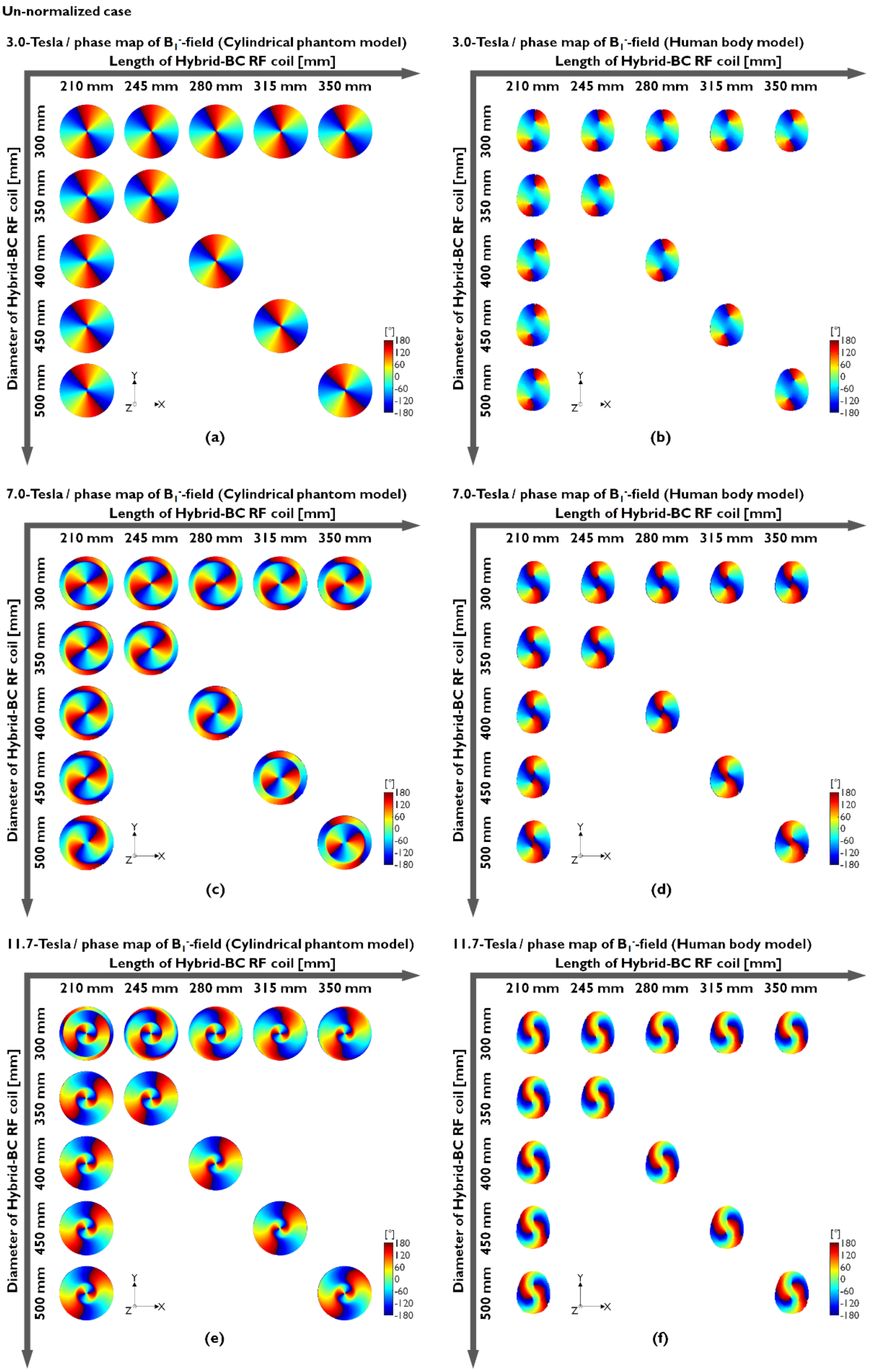

Figure S2. Phase map of the unnormalized B_1^--field distribution obtained when using a cylindrical phantom model (**a**, **c**, and **e**) or a human body model (**b**, **d**, and **f**).

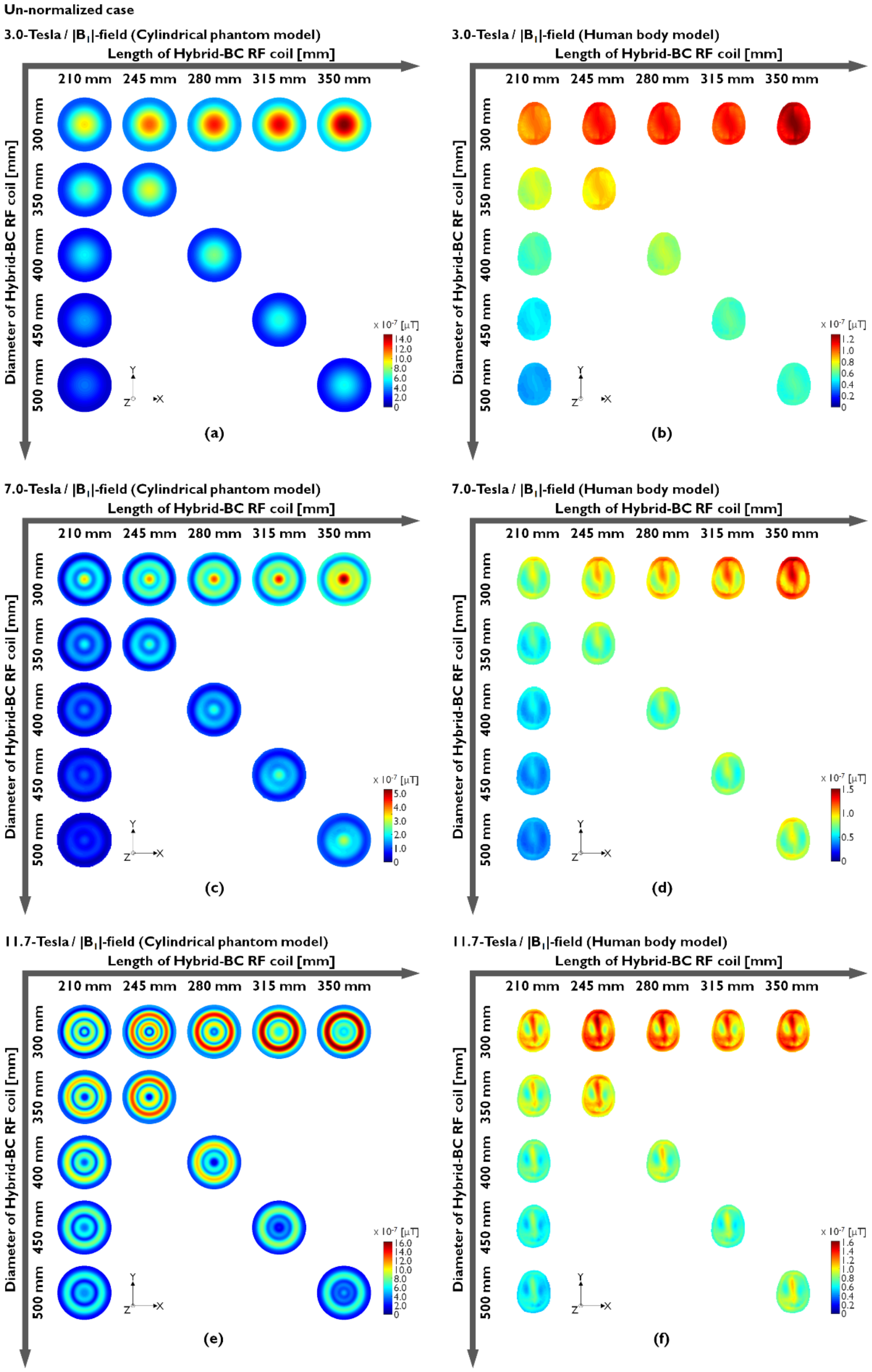

Figure S3. Unnormalized |B1|-field distribution obtained when using a cylindrical phantom model (**a**, **c**, and **e**) or a human body model (**b**, **d**, and **f**).

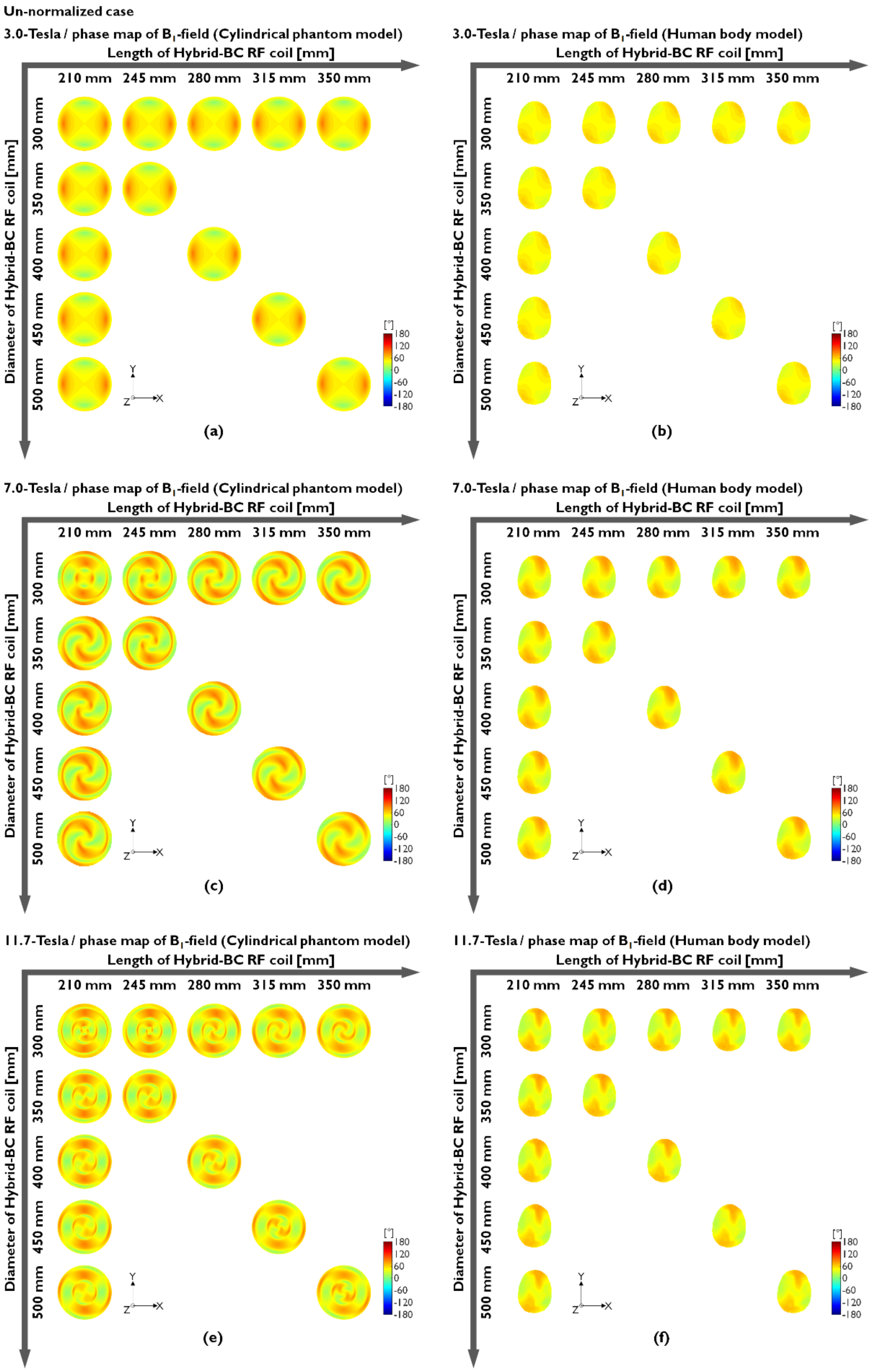

Figure S4. Phase map of the unnormalized B_1-field distribution obtained when using a cylindrical phantom model (**a**, **c**, and **e**) or a human body model (**b**, **d**, and **f**).

Un-normalized case

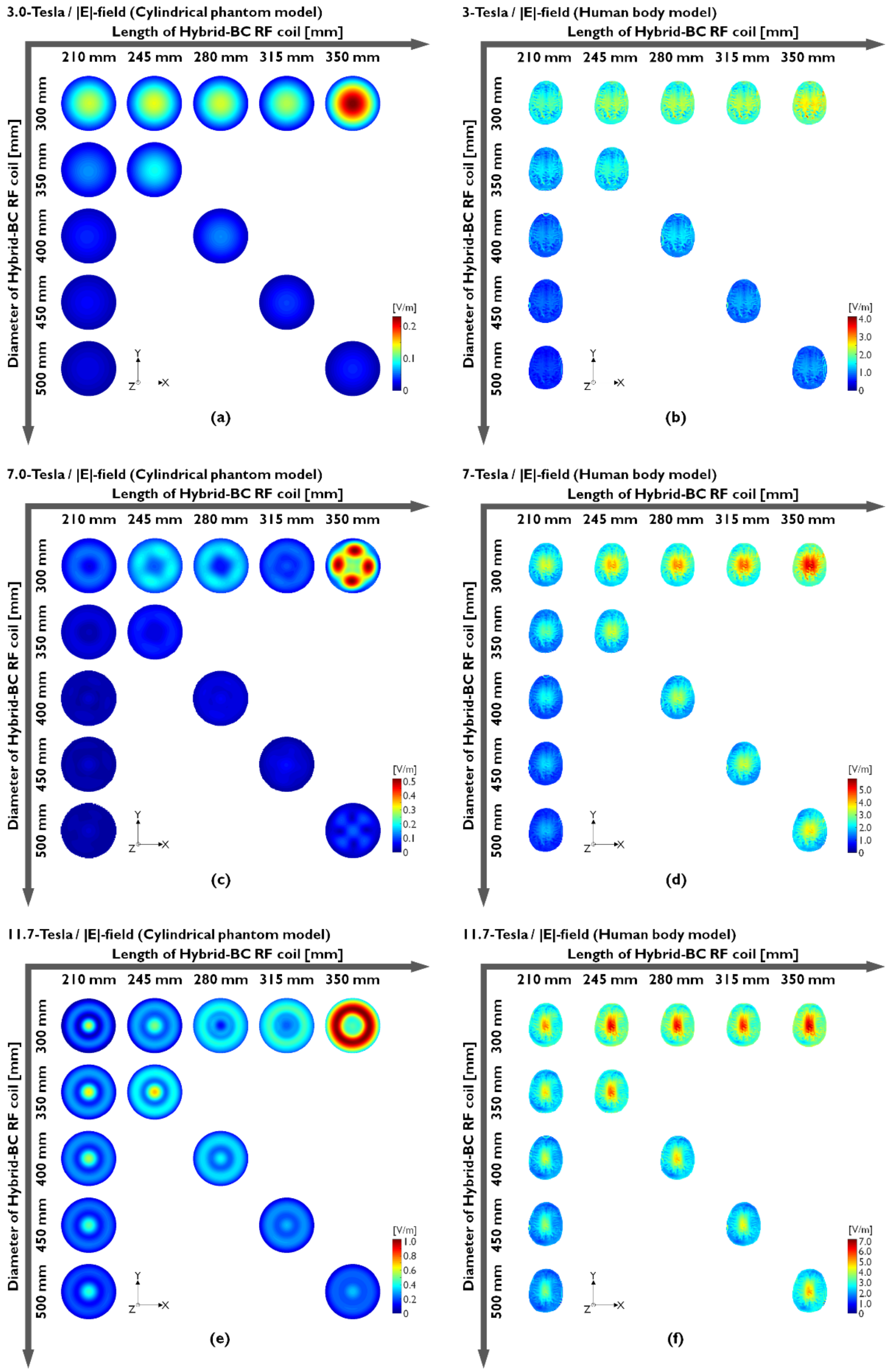

Figure S5. Unnormalized |E|-field distribution obtained when using a cylindrical phantom model (**a**, **c**, and **e**) or a human body model (**b**, **d**, and **f**).

Table S1. Unnormalized |B_1^-|-field values obtained when using a cylindrical phantom model or a human body model at a 3.0, 7.0, or 11.7 T MRI system.

Cylindrical phantom model (Distilled water)											
D (mm) \ L (mm)		Max values [×10⁻⁶ μT]					Mean values [×10⁻⁶ μT]				
		210	245	280	315	350	210	245	280	315	350
3.0 T	300	0.217	0.268	0.295	0.313	0.347	0.134	0.165	0.181	0.193	0.214
	350	0.160	0.200				0.100	0.124			
	400	0.121		0.166			0.076		0.103		
	450	0.095			0.139		0.059			0.086	
	500	0.076				0.130	0.047				0.080
7.0 T	300	0.106	0.148	0.160	0.154	0.155	0.055	0.076	0.084	0.085	0.090
	350	0.084	0.108				0.043	0.056			
	400	0.067		0.085			0.035		0.047		
	450	0.052			0.083		0.028			0.043	
	500	0.042				0.083	0.024				0.044
11.7 T	300	0.540	0.645	0.749	0.901	0.969	0.236	0.286	0.372	0.467	0.472
	350	0.571	0.693				0.284	0.350			
	400	0.519		0.629			0.273		0.334		
	450	0.439			0.512		0.234			0.274	
	500	0.377				0.436	0.201				0.229
Human body model (Duke phantom)											
D (mm) \ L (mm)		Max values [×10⁻⁶ μT]					Mean values [×10⁻⁶ μT]				
		210	245	280	315	350	210	245	280	315	350
3.0 T	300	0.020	0.023	0.024	0.024	0.027	0.011	0.013	0.013	0.013	0.015
	350	0.015	0.018				0.008	0.010			
	400	0.013		0.015			0.007		0.008		
	450	0.010			0.013		0.005			0.007	
	500	0.008				0.013	0.004				0.007
7.0 T	300	0.037	0.044	0.047	0.050	0.056	0.019	0.023	0.024	0.026	0.029
	350	0.028	0.034				0.015	0.018			
	400	0.024		0.034			0.012		0.018		
	450	0.020			0.035		0.010			0.018	
	500	0.018				0.039	0.009				0.020
11.7 T	300	0.061	0.074	0.072	0.067	0.069	0.032	0.038	0.036	0.033	0.034
	350	0.050	0.059				0.026	0.030			
	400	0.043		0.047			0.021		0.024		
	450	0.036			0.039		0.018			0.020	
	500	0.031				0.041	0.016				0.021

Table S2. Unnormalized |B1|-field values obtained when using a cylindrical phantom model or a human body model at a 3.0, 7.0, or 11.7 T MRI system.

Cylindrical phantom model (Distilled water)											
L (mm) / D (mm)		Center-point values [×10^{-6} μT]					Mean values [×10^{-6} μT]				
		210	245	280	315	350	210	245	280	315	350
3.0 T	300	0.963	1.169	1.277	1.352	1.500	0.478	0.590	0.652	0.694	0.775
	350	0.717	0.884				0.357	0.445			
	400	0.543		0.733			0.271		0.373		
	450	0.423			0.607		0.212			0.310	
	500	0.338				0.565	0.170				0.290
7.0 T	300	0.351	0.386	0.423	0.463	0.528	0.123	0.156	0.182	0.203	0.234
	350	0.200	0.236				0.091	0.115			
	400	0.141		0.224			0.074		0.109		
	450	0.115			0.250		0.063			0.117	
	500	0.101				0.291	0.055				0.134
11.7 T	300	0.779	0.787	0.505	0.598	0.657	0.545	0.691	0.769	0.871	0.845
	350	0.247	0.176				0.605	0.722			
	400	0.308		0.144			0.554		0.606		
	450	0.367			0.233		0.464			0.472	
	500	0.414				0.479	0.396				0.403
Human body model (Duke phantom)											
L (mm) / D (mm)		Center-point values [×10^{-6} μT]					Mean values [×10^{-6} μT]				
		210	245	280	315	350	210	245	280	315	350
3.0 T	300	0.101	0.116	0.117	0.118	0.132	0.099	0.111	0.111	0.110	0.123
	350	0.077	0.090				0.075	0.087			
	400	0.061		0.074			0.059		0.071		
	450	0.048			0.063		0.047			0.059	
	500	0.039				0.060	0.038				0.056
7.0 T	300	0.086	0.100	0.107	0.114	0.132	0.078	0.089	0.095	0.101	0.116
	350	0.067	0.081				0.060	0.072			
	400	0.055		0.078			0.050		0.070		
	450	0.047			0.081		0.043			0.072	
	500	0.043				0.089	0.038				0.080
11.7 T	300	0.114	0.133	0.127	0.118	0.127	0.111	0.131	0.127	0.119	0.128
	350	0.100	0.116				0.092	0.108			
	400	0.088		0.099			0.078		0.089		
	450	0.080			0.089		0.069			0.078	
	500	0.414				0.479	0.396				0.407

Table S3. Unnormalized |E|-field values obtained when using a cylindrical phantom model or a human body model at a 3.0, 7.0, or 11.7 T MRI system.

Cylindrical phantom model (Distilled water)											
L (mm) / D (mm)		Max values [×10^{-6} µT]					Mean values [×10^{-6} µT]				
		210	245	280	315	350	210	245	280	315	350
3.0 T	300	0.130	0.138	0.133	0.125	0.230	0.072	0.074	0.071	0.065	0.118
	350	0.060	0.088				0.036	0.049			
	400	0.034		0.058			0.022		0.032		
	450	0.027			0.040		0.016			0.022	
	500	0.022				0.034	0.013				0.018
7.0 T	300	0.119	0.201	0.194	0.118	0.519	0.084	0.146	0.139	0.089	0.271
	350	0.047	0.050				0.034	0.060			
	400	0.037		0.058			0.021		0.040		
	450	0.038			0.066		0.017			0.032	
	500	0.034				0.114	0.016				0.056
11.7 T	300	0.600	0.515	0.412	0.461	1.030	0.159	0.236	0.313	0.345	0.718
	350	0.657	0.702				0.227	0.310			
	400	0.559		0.382			0.224		0.271		
	450	0.470			0.309		0.194			0.208	
	500	0.428				0.322	0.168				0.183
Human body model (Duke phantom)											
L (mm) / D (mm)		Max values [×10^{-6} µT]					Mean values [×10^{-6} µT]				
		210	245	280	315	350	210	245	280	315	350
3.0 T	300	3.067	3.556	3.634	3.653	4.094	1.556	1.814	1.857	1.875	2.111
	350	3.295	3.993				1.203	1.421			
	400	2.696		2.600			0.947		1.179		
	450	1.766			2.258		0.762			0.999	
	500	1.520				2.195	0.623				0.959
7.0 T	300	3.793	4.568	4.821	5.074	5.867	2.184	2.610	2.756	2.906	3.352
	350	3.458	4.256				1.745	2.155			
	400	3.234		3.593			1.457		2.085		
	450	2.188			3.679		1.261			2.129	
	500	1.945				4.253	1.131				2.353
11.7 T	300	5.718	6.856	6.739	6.498	7.146	3.078	3.719	3.655	3.519	3.889
	350	5.001	5.953				2.643	3.175			
	400	4.447		5.787			2.322		2.727		
	450	4.047			4.710		2.113			2.486	
	500	4.135				5.380	2.020				2.870
